# FoxO3a Serves as a Biomarker of Oxidative Stress in Human Lens Epithelial Cells under Conditions of Hyperglycemia

**DOI:** 10.1371/journal.pone.0067126

**Published:** 2013-06-21

**Authors:** Ilangovan Raju, Krishnaswamy Kannan, Edathara C. Abraham

**Affiliations:** 1 Department of Biochemistry and Molecular Biology, University of Arkansas for Medical Sciences, Little Rock, Arkansas, United States of America; 2 Division of Rheumatology, Department of Internal Medicine, University of Arkansas for Medical Sciences, Little Rock, Arkansas, United States of America; Case Western Reserve University, United States of America

## Abstract

**Background:**

Forkhead box ‘O’ transcription factors (FoxOs) are implicated in the pathogenesis of type2 diabetes and other metabolic diseases. Abnormal activity of FoxOs was reported in the glucose and insulin metabolism. Expression of FoxO proteins was reported in ocular tissues; however their function under hyperglycemic conditions was not examined.

**Methods:**

Human lens epithelial cell line was used to study the function of FoxO proteins. Immunofluorescence, flow cytometry and Western blotting were employed to detect the FoxO proteins under the conditions of hyperglycemia.

**Results:**

In this study we examined the role of FoxO3a in hyperglycemia-induced oxidative stress in human lens epithelial cells. FoxO3a protein expression was elevated in a dose- and time-dependent fashion after high glucose treatment. Anti-oxidant defense mechanisms of the lens epithelial cells were diminished as evidenced from loss of mitochondrial membrane integrity and lowered MnSOD after 72 h treatment with high glucose. Taken together, FoxO3a acts as a sensitive indicator of oxidative stress and cell homeostasis in human lens epithelial cells during diabetic conditions.

**Conclusion:**

FoxO3a is an early stress response protein to glucose toxicity in diabetic conditions.

## Introduction

Type 2 Diabetes (T2D) is a metabolic disorder characterized by elevated high blood glucose levels due to relative insulin deficiency or insulin resistance [1]. Approximately 285 million people are affected by diabetes worldwide and this number is expected to increase in the coming years according to the International Diabetes Federation. The economic cost associated with diabetes is huge on individuals and healthcare delivery systems worldwide. In addition to age and obesity, hyperglycemia-induced oxidative stress is thought to be a major risk factor in the development of various diabetic complications that ultimately affect the well-being of eye, heart, kidney, nerves, and blood vessels [2], [3].

Hyperglycemia exerts its effects via several mechanisms including increased polyols pathway flux, increased formation of advanced glycation end products (AGE), activation of protein kinase C (PKC) and elevated levels of mitochondrial ROS in the affected tissues. In particular, increased oxidative stress and depleted anti-oxidant defenses by hyperglycemia are causally linked to mitochondrial dysfunction and increased ROS as well as micro and macrovasular complications [1], [4]. Growing evidence indicates that chronic exposure to hyperglycemic conditions accelerates the development of diabetic cataract [5], [6].

At present we lack a clear understanding of the critical components of glucose homeostasis as well as the precise molecular basis of diabetic cataract formation. Recently, Forkhead box O (FoxO) proteins, a family of transcription factors, have been implicated in the regulation of oxidative stress and several other diverse physiologic processes including stress resistance, cell differentiation, cell-cycle arrest, stem-cell homeostasis, metabolism, apoptosis, and life-span extension [7], [8]. In mammals, four isoforms of FoxOs and their molecular and protein characteristics have been reported [9], [10]. Of this, FoxO1 and FoxO3a share extensive homology and function and also have been implicated in the control of oxidative stress [11].

Abnormal activity of FoxOs has been implicated in the regulation of glucose and insulin metabolism as well as in the development of diabetes in a mouse model [12]–[14]. Expression of FoxO proteins in ocular tissues have been reported though its function has not been fully examined [15], [16]. In this study, we examined hyperglycemia-induced changes in FoxO3a protein expression, mitochondrial membrane integrity, and MnSOD, a transcriptional target of FoxO3a, to better understand the mechanisms of diabetic cataract in lens cells under *in vitro* conditions. Our results demonstrate that FoxO3a is a stress response protein in eye lens cells and its expression correlate with other surrogate biomarkers of oxidative stress such as mitochondrial membrane potential and MnSOD protein expression under conditions of hyperglycemia.

## Materials and Methods

### Cell Culture and Treatments

Human lens epithelial (HLE-B3) cell line was purchased from ATCC (Manassas, VA) and stock cultures were maintained in RPMI-1640 medium (Life Technologies, CA) supplemented with 20% FBS, penicillin/streptomycin antibiotic mix (50 U/mL) at 37°C in the presence of 5% CO_2_. At the time of experiment, cells from stock cultures centrifuged, washed, and seeded into 35 mm dishes for overnight in RPMI-1640 medium containing 10% FBS to minimize serum-mediated effect. In each dish, 1 million viable cells (1×10^6^) were seeded and treated with different doses of glucose for a period of 0–72 h. Mannitol treated (25 mM) group served as osmolality control. Cells were incubated at 37°C in CO_2_ incubator in humidified atmosphere for 0, 6, 12, 24, 48 and 72 h. In a separate experiment, cells were co-treated with 10 mM of anti-oxidant, N-acetyl cysteine (NAC) and harvested after 72 h.

### SDS-PAGE and Western Blot Analysis

After glucose treatment, cells were washed thrice with PBS and cell lysates were prepared in RIPA lysis buffer. Protein concentration was estimated by BCA assay and 20 µg of protein was loaded in each lane and bands were resolved by 10% SDS-PAGE. The gel was transferred to nitrocellulose membrane and blocked with 5% non-fat dry milk (NFDM) for an hour at room temperature. The blot was then incubated with any one of the FoxO antibodies for overnight at 4°C in 2.5% NFDM: anti-FoxO3a antibody (catalog # 2497), anti-phospho-FoxO1 (Thr 24)/phospho-FoxO3a (Thr32) antibody (catalog # 9464). All antibodies for Western blot detection were purchased from Cell Signaling (Danvers, MA, US). Blots were washed off primary antibody diluted with Tris-buffered-saline containing 0.1% tween-20 for three times and subsequently incubated with HRP-conjugated goat- anti- rabbit IgG antibody. Enhanced chemiluminscence substrate was used to detect the signal. To detect MnSOD specific protein, polyclonal rabbit anti-MnSOD (SOD2) antibody (catalog # 13533; Abcam, MA,) was used at a concentration of 1 in 5000. The blots were stripped and re-probed with mouse monoclonal α-tubulin antibody (Santa Cruz Biotechnology Inc., CA, catalog # sc5286) which served as an internal control.

### Measurement of Mitochondrial Membrane Potential (ΔΨm)

JC-1 is a cationic, positively charged fluorescent dye that exhibits potential-dependent accumulation in mitochondria, indicated by a fluorescence emission shift from green (∼525 nm) to red (∼590 nm). Consequently, the mitochondrial depolarization is indicated by a decrease in the red/green fluorescence intensity ratio. After appropriate treatment, cells were gently dislodged and loaded with 2 µM of JC-1 dye in 1 mL PBS and incubated for l5 min at 37°C. Cells were immediately analyzed by a flow cytometer (BD FACS LSR II) and were gated using appropriate settings in FL1 (green) and FL2 (red) channels. As a positive control, cells were treated with the mitochondrial membrane disrupter, CCCP (carbonyl cyanide 3-chlorophenylhydrazone (50 µM) for 5 min at 37°C. For each treatment condition, at least 10,000 cells were gated and statistically analyzed.

### Immunofluorescence Staining for FoxO3a

For confocal microscopy, 20,000 cells were plated and treated with low and high glucose for 72 hours in 35 mm glass-bottom culture dishes (Mat-Tek, MA). At indicated time intervals, cells were washed in PBS and fixed with 4% paraformaldehyde for 20 min at room temperature and then permeabilized with 0.3% Triton X-100 for 10 minutes. After permeabilization, cells were washed with PBS, blocked with 10% normal goat serum and stained with anti-FoxO3a antibody in blocking buffer for overnight at 4°C at a dilution of 1 in 100. Cells were subsequently stained with goat-anti-rabbit conjugated Alexa Flour 488 (Life Technologies, CA) at a dilution of 1 in 500 in blocking buffer for 1 h at room temperature. The nucleus was counter-stained with Hoechst 33342. Cells were immediately examined under LSM 510 confocal microscope and the images were captured at x63 oil immersion. The cytoplasmic and nuclear localization of FoxO3a was determined by counting at least 100 cells per experiment. These experiments were repeated at least three times and the results were expressed as percentage of positive cells.

### Statistical Analyses

One way ANOVA followed by post hoc comparisons by Tukey test were made to determine the statistical significance between various treated groups. All tests were performed using Prism 4 software (Graphpad, CA). The results were expressed as mean ± SD. The *p* values <0.05 were considered significant.

## Results

### Spatial and Temporal Changes in the Expression of FoxO3a

**Figure 1:**
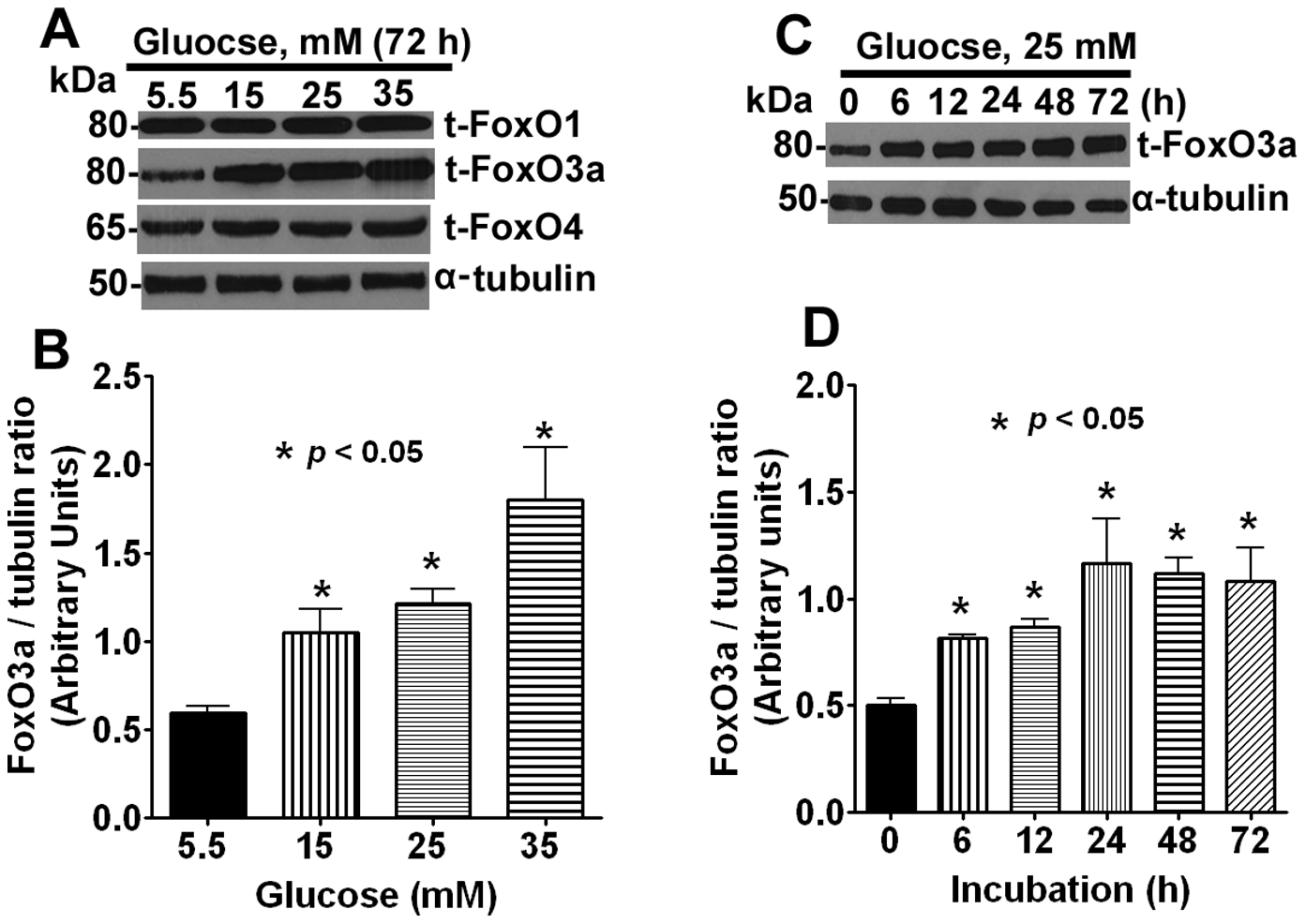
Hyperglycemic condition induces FoxO3a expression in dose and time-dependent manner. **A**. Representative Western blots data showing the expression of FoxO3a protein after glucose treatment. Human lens epithelial cells were treated with different doses of glucose and lysates were prepared after 72 h. Glucose at 5.5 mM served as basal concentration, whereas other doses were of hyperglycemic. **B**. Densitometric data of FoxO3a expression was determined by Image J software (NIH, USA) from three independent experiments. The α-tubulin served as loading control. A dose-dependent increase in FoxO3a expression was observed. The error bar indicates mean ± SD. **C**. Representative Western blots data showing the expression of FoxO3a at different time points after glucose treatment. Cells were treated with 25 mM concentration of glucose and cell lysates were prepared at 0, 6, 12, 24, 48 and 72 h. **D**. Densitometric data of FoxO3a for the Western blot obtained in three independent experiments. A time-dependent increase in FoxO3a expression was observed.

To investigate the role of FoxO3a in human lens epithelial cells under the influence of high glucose, we first examined the spatial and temporal expression of this protein. Initially, cells were treated with different concentrations of glucose ranging from 5.5 to 35 mM for 72 h and then we examined the expression of three FoxO proteins – FoxO1, FoxO3a and FoxO4. These experiments revealed that only FoxO3a was highly responsive to hyperglycemic treatment whereas no appreciable changes were detected with FoxO1 and FoxO4 (Figure 1A & 1B).

We observed that glucose as low as 15 mM concentration showed a statistically significant increase in protein expression and this trend continued up to 35 mM. Further experiments were then carried out using 5.5 mM as a basal control (low glucose) and 25 mM as high glucose concentration (or hyperglycemic dose). We then investigated the effect of 25 mM high glucose at various time points starting 0–72 h and compared FoxO3a expression with low glucose concentration (5.5 mM). Our results revealed that FoxO3a was up-regulated as early as 6 h post-exposure which was statistically significant from control cells and this effect persisted at all the time points during a 0–72 h period (Figure 1C & 1D).

### N-acetyl Cysteine Reverses the FoxO3a Expression

**Figure 2:**
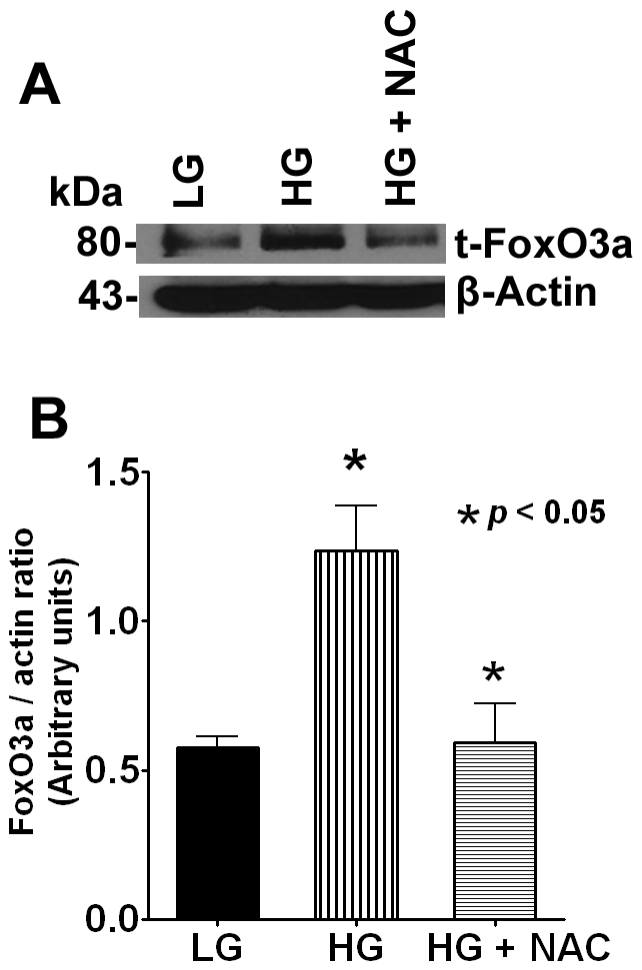
NAC treatment reverses the activation of FoxO3a. **A**. Cells were treated with 25 mM glucose+NAC for 72 h and the Western blot probed with FoxO3a antibody. Representative Western blots data showing FoxO3a protein expression. **B**. Densitometric analysis of the Western blot obtained in three separate experiments. Data shows NAC-mediated protective effect against oxidative stress by glucose. The β-actin was shown as loading control.

Based on our previous results we reasoned that high glucose exerts oxidative stress in eye lens cells. To neutralize this adverse effect, we co-incubated cells with and without NAC to study the protective effect of NAC on FoxO3a expression at 72 h time point. Our results suggest that NAC abrogated the high glucose-mediated FoxO3a up-regulation as shown in Figure 2.

### Subcellular Localization of FoxO3a Protein

**Figure 3:**
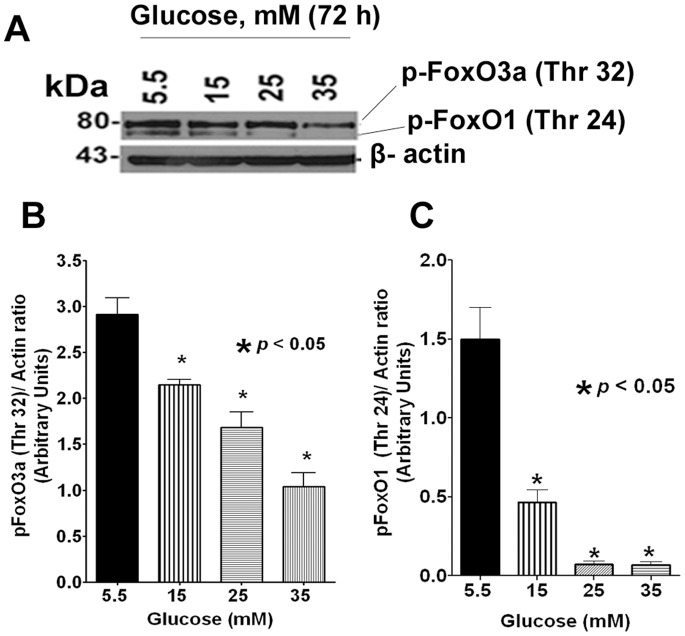
Western blot analysis of phospho-FoxO1 (Thr 24)/phospho-FoxO3a (Thr 32). **A**. A typical Western blot image of pFoxO1 (Thr 24)/pFoxO3a (Thr 32) protein expression after glucose treatment for 72 h. **B and C**. Densitometric analysis of the Western blot from three independent experiments. Data showed that both pFoxO1 (Thr 24) and pFoxO3a (Thr 32) were significantly dephosphorylated under hyperglycemic conditions in a dose-dependent manner. The β-actin was shown as loading control.

**Figure 4:**
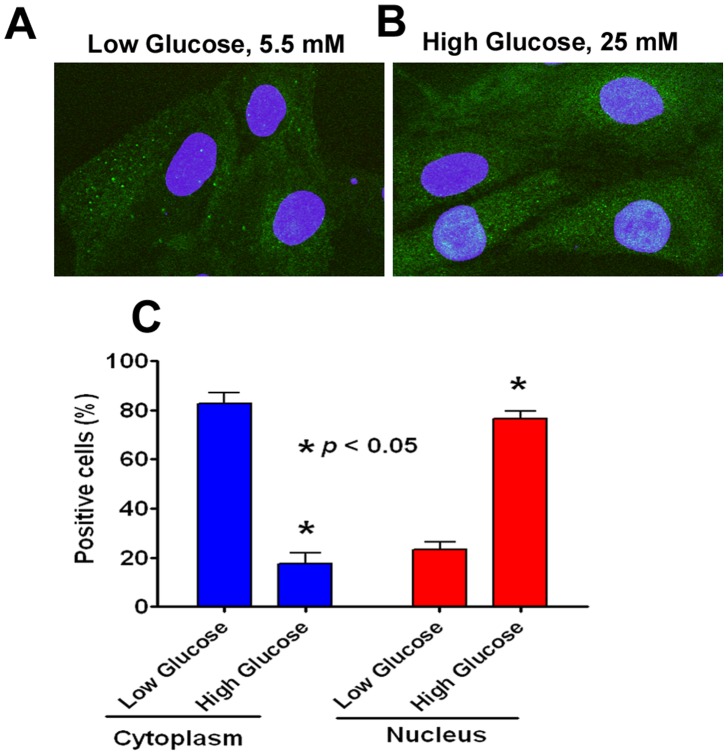
Subcellular localization of FoxO3a. A. Representative confocal image showing FoxO3a expression in the cytoplasm under low basal concentration of glucose. **B**. Representative confocal image showing enhanced translocation of FoxO3a under hyperglycemic conditions. **C**. Quantification of immunofluorescence signal for FoxO3a in the cytosol and nucleus.

The fact that subcellular localization of FoxO transcription factors is critical for various cellular functions have been extensively reported [17], [18]. Under steady state conditions, Akt/PKB phosphorylation of FoxO excludes it from the nucleus by binding to 14-3-3-protein in the cytosol [17]. Under oxidative stress, translocation of FoxO proteins occurs either by stress kinase JNK pathway or by dysregulation of PP2A phosphatases [19]. We therefore asked to determine whether high glucose mediates FoxO3a translocation to nucleus by one of the aforementioned pathways which were studied by Western blotting. Our results showed that levels of both p-FoxO1 (Thr 24)/p-FoxO3a (Thr 32) were dephosphorylated in a dose-dependent manner at 72 h time period (Figure 3). To extend these findings and to confirm nuclear localization of FoxO3a, we also examined subcellular localization of FoxO3a by confocal microscopy after glucose treatment at 72 h time point. Our results showed that 70% of cells were immunostaining positive for FoxO3a within the nucleus due to high glucose treatment, whereas this occurred in only 20% of cells when treated with low glucose (Figure 4).

### Hyperglycemia-induced Changes in MnSOD

**Figure 5:**
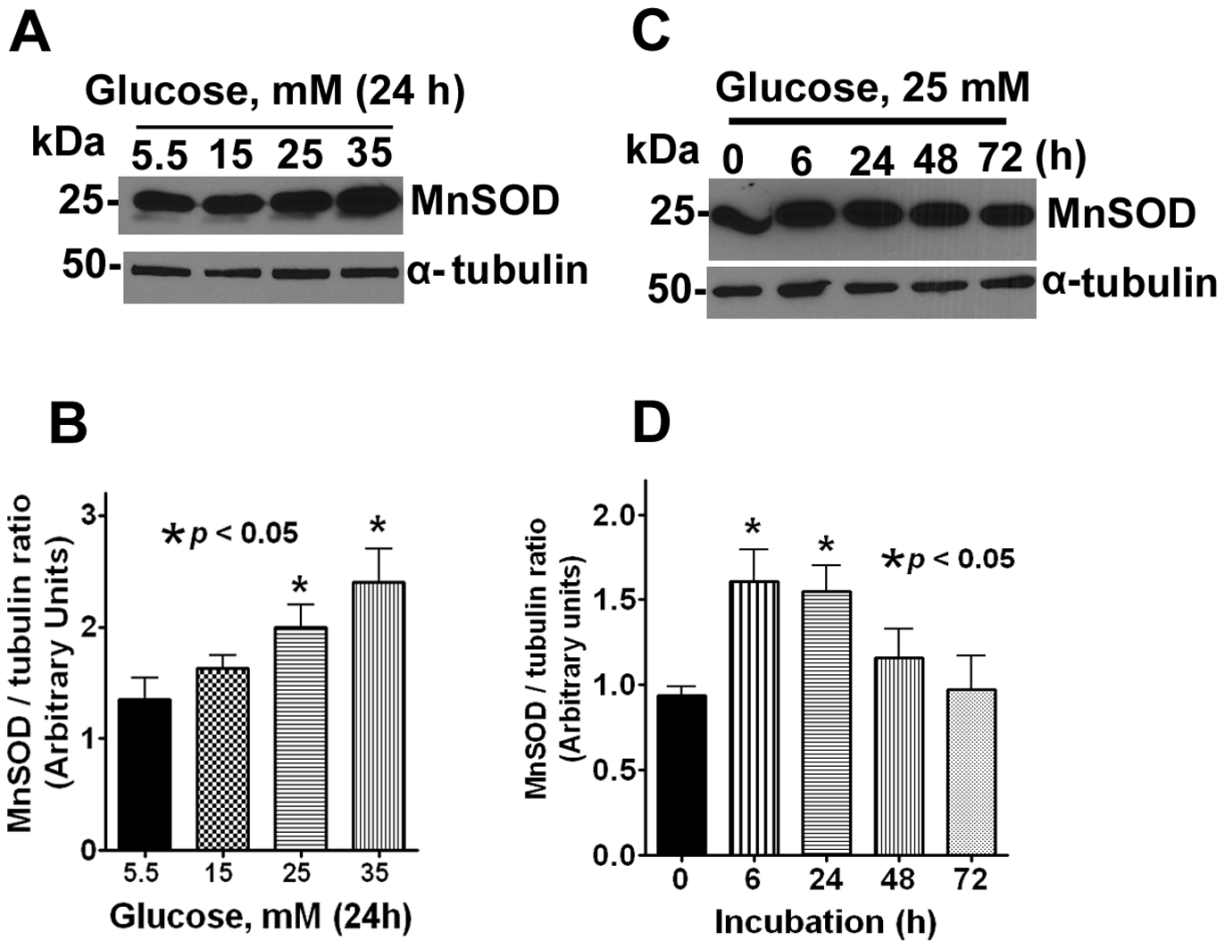
Hyperglycemic conditions diminished the expression of MnSOD. **A.** Representative Western blots data showing the expression of MnSOD in lens epithelial cells treated with different doses of glucose for 24 h. The α-tubulin served as loading control. **B**. Densitometric analysis of the Western blot obtained in three independent experiments. The results are expressed as mean ± SD. **C**. Representative Western blots showing the expression of MnSOD in lens epithelial cells treated with 25 mM of glucose at different time points. The α-tubulin served as loading control. **D**. Densitometric analysis of the Western blot obtained in three independent experiments.

MnSOD and catalase are the major anti-oxidant enzymes expressed in normal human lens epithelial cells which are transcriptional targets of FoxO1 and FoxO3a [32]–[33]. To determine whether MnSOD protein expression was altered under hyperglycemic conditions, we assessed the expression of MnSOD by protein gel analysis. We observed a dose-dependent increase in MnSOD expression after 24 h incubation with high glucose (Figure 5A–B). In addition, our data revealed a biphasic response of MnSOD protein expression during which there was a significant increase during the first 6 h period followed by a gradual decrease in expression between 24–72 h suggesting an adaptive responsive during the early hours of treatment followed by dysregulation of anti-oxidant biochemical defenses (Figure 5C–D).

### Loss of Mitochondrial Membrane Integrity

**Figure 6:**
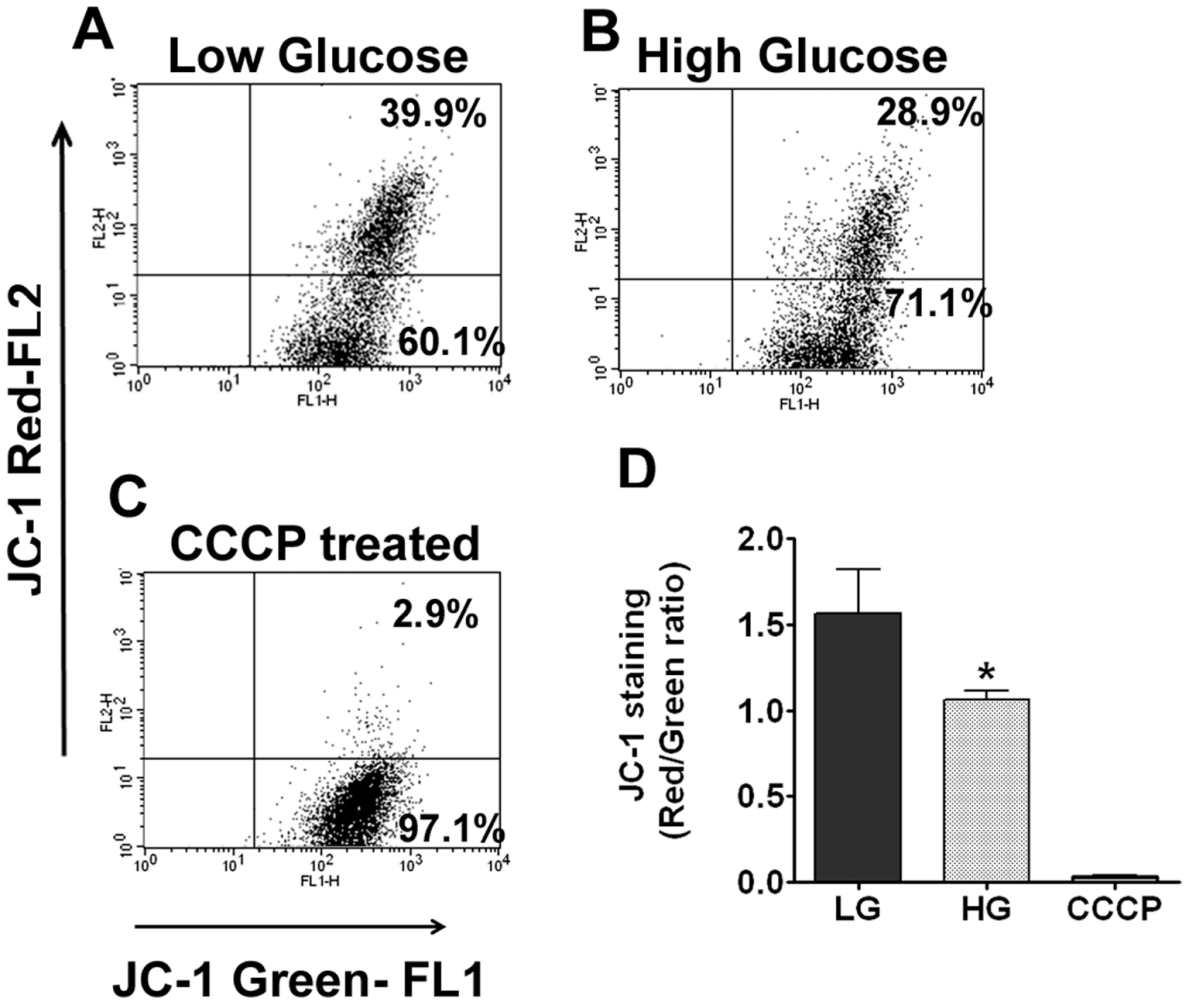
Hyperglycemic conditions decrease the mitochondrial membrane integrity. **A–C**: Representative flow cytometry histogram showing JC-1 staining after treatment with low glucose, high glucose and CCCP-treatment (positive control). **D**. Graph showing significant reduction of mitochondrial membrane integrity in cells treated with high glucose. The arbitrary units indicate the ratio of red/green fluorescence. The shift from red to green indicates the mitochondrial membrane depolarization.

Oxidative stress due to mitochondrial dysfunction was evaluated by JC-1 staining which measures mitochondrial membrane potential and integrity. JC-1 forms red aggregates in healthy and intact mitochondria whereas this becomes JC-1 monomers in the cytoplasm when the mitochondrial membrane integrity is compromised [15]. Lens epithelial cells were treated with both low and high glucose for 72 h and analyzed for JC-1 staining by flow cytometry. The data included in the flow cytometry histogram (Figure 6) demonstrated an adverse effect on mitochondrial membrane integrity as evidenced from the shift in the red fluorescence to green suggesting glucose-mediated toxicity.

## Discussion

Hyperglycemia, characteristic of T2D is associated with the development of serious metabolic complications including cataracts, atherosclerosis, and nephropathy [1], [20], [21]. Prior studies have suggested that oxidative stress plays a key role in the development of diabetic cataract in which reactive oxygen species (ROS) are a primary cause of cellular damage [8], [28]. Studies from our laboratory have shown that the chaperone function of α-crystallin was significantly diminished during diabetic conditions [22], [23]. However, the molecular determinants of oxidative stress and the sequence of biochemical events leading to loss of α-crystallin chaperone activity in lens cells are far from understood [21], [24].

In this study, we demonstrate that FoxO3a is an early biomarker of oxidative stress in human eye lens cells under conditions of hyperglycemia. Elevated levels of FoxO3a expression was detected in eye lens cells as early as six hours after high glucose treatment which persisted throughout the incubation period which were both dose- and time-dependent. We also demonstrated that MnSOD, anti-oxidant enzyme and a transcriptional target of FoxO3a was dysregulated in these cells. In addition, we observed that high glucose significantly reduced mitochondrial membrane integrity suggesting mitochondria could be one of the targets of hyperglycemia in lens cells. Together, these data provide a mechanistic insight into the underlying causes of oxidative stress in eye lens cells where FoxOs seem to play a cell homeostatic role.

One of the goals of our study was to identify and characterize the early molecular determinants of oxidative stress after high glucose exposure so that they can be modulated by novel therapies so as to preserve the chaperone activity of α-crystallin proteins. Since FoxOs regulate the expression of anti-oxidant enzymes, MnSOD, catalase and stress-related gene products [25], [26], they could serve as good candidate proteins and bio-indicators of oxidative stress in these lens cells.

FoxO1, FoxO3a and FoxO4 were shown to be involved in glucose metabolism in organ systems and experimental models (reviewed in [14], [17], [27]). Of the three FoxOs investigated in the present study, we found that only FoxO3a responded to high glucose treatment in the lens cells. Thus, we elected to study the role of FoxO3a in diabetic cataract by using a human eye lens cells.

Importantly, the seminal functions of FoxOs are mainly revealed under stress conditions, for example in a diabetic background [17], [28]. This notion is corroborated by the conditional deletion of FoxO1, FoxO3 or FoxO4 in adult mice where only a modest increase in neoplastic phenotype is observed [29], whereas, under adverse conditions, DAF-16/FoxO in C.*elegans* enable worms to enter into ‘dauer’ stage for resistance of oxidative stress and long term survival. Taken together, this argues that the main, if not only, role of FoxOs is to act as homeostasis regulators, particularly in response to stress [30].

Generally, the effector functions of FoxOs are mediated by two evolutionarily conserved signalling pathways. In the presence of growth factors, FoxOs are negatively regulated by the canonical insulin signalling pathway through PI3K and protein kinase B (PKB; also known as AKT) which results in sequestration of FoxOs in the cytosol binding to 14-3-3 protein in an inactive state by phosphorylation [31]–[33]. Under conditions of oxidative stress, FoxOs are activated through Jun N-terminal kinase (JNK) signalling pathway which result in nuclear translocation and DNA binding [31]–[33]. In addition, several other signalling pathways are known to modulate FoxO activity [30]. In particular, immediately upstream of FoxO, the activity of Akt itself is governed by several protein kinases and phosphatases [34]. Taken together, PI3K-PKB-FoxO signaling pathway ensures longer lifespan, whereas, the deregulation of this pathway contributes to two major age-related diseases, namely cancer and diabetes suggesting a pivotal role for FoxOs.

Given its importance in cell homeostasis, FoxO-mediated regulation of its transcriptional targets *in vivo* is highly context-specific, even in the same cell type [29]. In the present study, we demonstrated nuclear translocation of FoxO3a after high glucose suggesting a role for JNK signaling pathway. Further studies are needed to elucidate the role of JNK and other signaling cascades. Our data revealed a biphasic response of MnSOD protein expression suggesting anti-oxidant biochemical defense during the first 0–24 h which diminished thereafter suggesting exposure to high glucose for a period beyond 48 h overwhelmed the anti-oxidant capacity of the cell. Results from two independent studies have suggested use of anti-oxidants prevented the oxidative damage to lens epithelial cells and arrested the loss of chaperone-like activity of α-crystallin in rat lens [24], [35]. In agreement, we also demonstrated restoration of FoxO3a protein expression to base levels with NAC, a well-known anti-oxidant suggesting dysregulation of FoxO3a expression can be reversed with the use of anti-oxidants.

In conclusion, our results demonstrate that exposure of lens epithelial cells to high glucose led to increased oxidative stress as evidenced from loss of mitochondrial membrane integrity and increased expression of FoxO3a which was reversed by NAC. Anti-oxidant enzyme MnSOD was modulated by high glucose in a biphasic manner suggesting the modulation of anti-oxidant enzymes by FoxO3a may be context-specific. To our knowledge, this is the first study reporting dysregulation of FoxO activity in lens cells under conditions of hyperglycemia. Further studies are necessary to elucidate the regulation of FoxO3a-mediated transcriptional activity during various stages of diabetic cataract development. This may be achieved by dissecting out the components of the signaling events during hyperglycemia induced metabolic stress.

## References

[1] Choi SW, Benzie IF, Ma SW, Strain JJ, Hannigan BM (2008) Acute hyperglycemia and oxidative stress: direct cause and effect? Free Radic Biol Med 44: 1217–1231. 18226604 10.1016/j.freeradbiomed.2007.12.005

[2] WHO website. Available:http://www.who.int/diabetes/facts/world_figures/en/2010. Accessed 2013 May 23.

[3] Srivastava SK, Ansari NH, Bhatnagar A (1990) Sugar induced cataractogenesis: a paradigm of oxidative tissue pathology? Lens Eye Toxic Res 7: 161–171. 2275929

[4] Rains JL, Jain SK (2011) Oxidative stress, insulin signaling, and diabetes. Free Radic Biol Med 50: 567–575. 21163346 10.1016/j.freeradbiomed.2010.12.006 PMC3557825

[5] Argirova M, Breipohl W (2002) Comparison between modifications of lens proteins resulted from glycation with methylglyoxal, glyoxal, ascorbic acid, and fructose. J Biochem Mol Toxicol 16: 140–145. 12112714 10.1002/jbt.10031

[6] Ughade SN, Zodpey SP, Khanolkar VA (1998) Risk factors for cataract: a case control study. Indian J Ophthalmol 46: 221–227. 10218305

[7] Kops GJ, Dansen TB, Polderman PE, Saarloos I, Wirtz KW, et al (2002) Forkhead transcription factor FOXO3a protects quiescent cells from oxidative stress. Nature 419: 316–321. 12239572 10.1038/nature01036

[8] Murphy CT, McCarroll SA, Bargmann CI, Fraser A, Kamath RS, et al (2003) Genes that act downstream of DAF-16 to influence the lifespan of Caenorhabditis elegans. Nature 424: 277–283. 12845331 10.1038/nature01789

[9] Calnan DR, Brunet A (2008) The FoxO code. Oncogene 27: 2276–2288. 18391970 10.1038/onc.2008.21

[10] Greer EL, Brunet A (2005) FOXO transcription factors at the interface between longevity and tumor suppression. Oncogene 24: 7410–7425. 16288288 10.1038/sj.onc.1209086

[11] Essers MA, Weijzen S, de Vries-Smits AM, Saarloos I, de Ruiter ND, et al (2004) FOXO transcription factor activation by oxidative stress mediated by the small GTPase Ral and JNK. EMBO J 23: 4802–4812. 15538382 10.1038/sj.emboj.7600476 PMC535088

[12] Kim DH, Zhang T, Lee S, Dong HH (2013) FoxO6 in Glucose Metabolism. J Diabetes. 10.1111/1753-0407.12027 PMC3657578 23324123

[13] Kousteni S (2012) FoxO1, the transcriptional chief of staff of energy metabolism. Bone 50: 437–443. 21816244 10.1016/j.bone.2011.06.034 PMC3228887

[14] Nakae J, Biggs WH III, Kitamura T, Cavenee WK, Wright CV, et al (2002) Regulation of insulin action and pancreatic beta-cell function by mutated alleles of the gene encoding forkhead transcription factor Foxo1. Nat Genet 32: 245–253. 12219087 10.1038/ng890

[15] Li G, Luna C, Navarro ID, Epstein DL, Huang W, Gonzalez P, et al (2011) Resveratrol prevention of oxidative stress damage to lens epithelial cell cultures is mediated by forkhead box O activity. Invest Ophthalmol Vis Sci 52: 4395–4401. 21345980 10.1167/iovs.10-6652 PMC3175971

[16] Zheng T, Lu Y (2011) Changes in SIRT1 expression and its downstream pathways in age-related cataract in humans. Curr Eye Res 36: 449–455. 21501079 10.3109/02713683.2011.559301

[17] Ponugoti B, Dong G, Graves DT (2012) Role of forkhead transcription factors in diabetes-induced oxidative stress. Exp Diabetes Res 2012: 939751 doi: 10.1155/2012/939751 22454632 10.1155/2012/939751 PMC3290826

[18] Kowluru A, Matti A (2012) Hyperactivation of protein phosphatase 2A in models of glucolipotoxicity and diabetes: potential mechanisms and functional consequences. Biochem Pharmacol 84: 591–597. 22583922 10.1016/j.bcp.2012.05.003

[19] Singh A, Ye M, Bucur O, Zhu S, Tanya SM, et al (2010) Protein phosphatase 2A reactivates FOXO3a through a dynamic interplay with 14-3-3 and AKT. Mol Biol Cell 21: 1140–1152. 20110348 10.1091/mbc.E09-09-0795 PMC2836964

[20] Obrosova IG, Chung SS, Kador PF (2010) Diabetic cataracts: mechanisms and management. Diabetes Metab Res Rev 26: 172–180. 20474067 10.1002/dmrr.1075

[21] Pollreisz A, Schmidt-Erfurth U (2010) Diabetic cataract-pathogenesis, epidemiology and treatment. J Ophthalmol 2010: 608751 doi: 10.1155/2010/608751 20634936 10.1155/2010/608751 PMC2903955

[22] Cherian M, Abraham EC (1995) Diabetes affects alpha-crystallin chaperone function. Biochem Biophys Res Commun 212: 184–189. 7612005 10.1006/bbrc.1995.1954

[23] Thampi P, Zarina S, Abraham EC (2002) alpha-Crystallin chaperone function in diabetic rat and human lenses. Mol Cell Biochem 229: 113–118. 11936835 10.1023/a:1017980713089

[24] Kumar PA, Suryanarayana P, Reddy PY, Reddy GB (2005) Modulation of alpha-crystallin chaperone activity in diabetic rat lens by curcumin. Mol Vis 11: 561–568. 16088325

[25] Nemoto S, Finkel T (2002) Redox regulation of forkhead proteins through a p66shc-dependent signaling pathway. Science 295: 2450–2452. 11884717 10.1126/science.1069004

[26] Tran H, Brunet A, Griffith EC, Greenberg ME (2003) The many forks in FOXO’s road. Sci STKE 2003: RE5 10.1126/stke.2003.172.re5 [doi];2003/172/re5 [pii]. 12621150 10.1126/stke.2003.172.re5

[27] Haeusler RA, Kaestner KH, Accili D (2010) FoxOs function synergistically to promote glucose production. J Biol Chem 285: 35245–35248. 20880840 10.1074/jbc.C110.175851 PMC2975147

[28] Nakae J, Oki M, Cao Y (2008) The FoxO transcription factors and metabolic regulation. FEBS Lett 582: 54–67. 18022395 10.1016/j.febslet.2007.11.025

[29] Paik JH, Kollipara R, Chu G, Ji H, Xiao Y, et al (2007) FoxOs are lineage-restricted redundant tumor suppressors and regulate endothelial cell homeostasis. Cell 128: 309–323. 17254969 10.1016/j.cell.2006.12.029 PMC1855089

[30] Eijkelenboom A, Burgering BM (2013) FOXOs: signalling integrators for homeostasis maintenance. Nat Rev Mol Cell Biol 14: 83–97. 23325358 10.1038/nrm3507

[31] Dansen TB (2011) Forkhead Box O transcription factors: key players in redox signaling. Antioxid Redox Signal 14: 559–561. 21083421 10.1089/ars.2010.3778

[32] de Keizer PL, Burgering BM, Dansen TB (2011) Forkhead box O as a sensor, mediator, and regulator of redox signaling. Antioxid Redox Signal 14: 1093–1106. 20626320 10.1089/ars.2010.3403

[33] Tzivion G, Dobson M, Ramakrishnan G (2011) FoxO transcription factors; Regulation by AKT and 14–3-3 proteins. Biochim Biophys Acta 1813: 1938–1945. 21708191 10.1016/j.bbamcr.2011.06.002

[34] Ni YG, Wang N, Cao DJ, Sachan N, Morris DJ, et al (2007) FoxO transcription factors activate Akt and attenuate insulin signaling in heart by inhibiting protein phosphatases. Proc Natl Acad Sci U S A 104: 20517–20522. 18077353 10.1073/pnas.0610290104 PMC2154463

[35] Kumar PA, Reddy PY, Srinivas PN, Reddy GB (2009) Delay of diabetic cataract in rats by the antiglycating potential of cumin through modulation of alpha-crystallin chaperone activity. J Nutr Biochem 20: 553–562. 18789666 10.1016/j.jnutbio.2008.05.015

